# A Global Phylogeny of Leafmining *Ectoedemia* Moths (Lepidoptera: Nepticulidae): Exploring Host Plant Family Shifts and Allopatry as Drivers of Speciation

**DOI:** 10.1371/journal.pone.0119586

**Published:** 2015-03-18

**Authors:** Camiel Doorenweerd, Erik J. van Nieukerken, Steph B. J. Menken

**Affiliations:** 1 Department of Terrestrial Zoology, Naturalis Biodiversity Center, Leiden, The Netherlands; 2 Institute for Biodiversity and Ecosystem Dynamics, University of Amsterdam, Amsterdam, The Netherlands; University of Arkansas, UNITED STATES

## Abstract

**Background:**

Host association patterns in *Ectoedemia* (Lepidoptera: Nepticulidae) are also encountered in other insect groups with intimate plant relationships, including a high degree of monophagy, a preference for ecologically dominant plant families (e.g. Fagaceae, Rosaceae, Salicaceae, and Betulaceae) and a tendency for related insect species to feed on related host plant species. The evolutionary processes underlying these patterns are only partly understood, we therefore assessed the role of allopatry and host plant family shifts in speciation within *Ectoedemia*.

**Methodology:**

Six nuclear and mitochondrial DNA markers with a total aligned length of 3692 base pairs were used to infer phylogenetic relationships among 92 species belonging to the subgenus *Ectoedemia* of the genus *Ectoedemia*, representing a thorough taxon sampling with a global coverage. The results support monophyletic species groups that are congruent with published findings based on morphology. We used the obtained phylogeny to explore host plant family association and geographical distribution to investigate if host shifts and allopatry have been instrumental in the speciation of these leafmining insects.

**Significance:**

We found that, even though most species within species groups commonly feed on plants from one family, shifts to a distantly related host family have occasionally occurred throughout the phylogeny and such shifts are most commonly observed towards Betulaceae. The largest radiations have occurred within species groups that feed on Fagaceae, Rosaceae, and Salicaceae. Most species are restricted to one of the seven global biogeographic regions, but within species groups representatives are commonly found in different biogeographic regions. Although we find general patterns with regard to host use and biogeography, there are differences between clades that suggest that different drivers of speciation, and perhaps drivers that we did not examine, have shaped diversity patterns in different clades.

## Introduction

Insect herbivores constitute the most species-rich group of insects and more than half of the world’s known species are insects [1]. However, how evolutionary processes in insect-plant networks have shaped radiations in time and space is yet little understood [2–5]. As phylogenetic studies of insects as well as plants are becoming more complete in terms of taxon sampling, more robust by including more genetic markers and better dated with molecular clock methods, a complete picture of species interaction patterns as well as a more complete understanding of the relative importance of the various evolutionary factors that drive herbivorous insect radiations is coming within reach [5–7]. The two most salient characteristics of plant-insect associations were unveiled long before the phylogenetics era, but were recently corroborated by molecular studies, viz. 1) related insects tend to feed on related plants and 2) insect diet breadth decreases as interactions become more intimate, such as with internal feeding [3,8]. Yet, strict co-cladogenesis between insects and their plant hosts is rare, and occasional host shifts towards distantly related plants occur throughout most groups [2,9].

Multiple phylogenetic studies that incorporated molecular clock analyses have shown that contemporaneous parallel cladogenesis has not been common in the evolution of plants and their herbivorous insects [10–15]. Also little evidence has been found for ‘asynchronous parallel cladogenesis’, where plants diversified first and herbivores later mirrored the speciation events of their hosts through resource tracking [16]. Instead a ‘resource archipelago’ scenario seems to be realistic [17]. In this scenario, plants have radiated independently from herbivores. Herbivore radiation on the plant hosts occurred later, at least partly through ecological speciation and, as a result, the herbivore phylogeny mirrors the plant phylogeny only in part. During ecological speciation new species arise after diverging in resource use, which for herbivores is commonly a dietary change, and this may happen in sympatry or allopatry [18–20]. It has been argued quite a few times in the past century, however, that ecological factors driving speciation are often overestimated, whereas geographic factors are underestimated [16,21,22].

Phylogenies can be used to differentiate between the two speciation scenarios: in cases of ecological speciation, there will be resource-shifts between sister-species, but if allopatric speciation is the rule, the resources will remain the same [17]. Food resources for phytophagous insects constitute many different resource dimensions. Differences occur for example within a single plant between tissue types and developmental stages, between individual plants, between different parts of a plant population, within a single plant species in different seasons or between species [17]. Research on ecological speciation has long focussed on plant secondary metabolites, and the ability to digest and/or detoxify these by insects. For some groups in which both the herbivore and plant evolution has been studied in detail, plant chemistry has indeed been shown to drive speciation, at least partly, in both the insect and the plant [23,24]. However, it is also becoming increasingly clear that for many groups of herbivores a wider range of resource dimensions may drive speciation [17,18].

Changes in feeding mode or changes in host use are some of the evolutionary events of an herbivorous insect species that involve different resource dimensions. Feeding mode is the term loosely defining the combination of the plant part that is used (e.g. leaf, stem, fruit) and the method of residing in, or consuming, the plant tissue (e.g. external feeding, galling, mining). The feeding mode is constrained by morphological traits of the insect such as the shape and functionality of the mouthparts, but also for example the shape of the ovipositor, of which the dimensions determine the plant tissue that can be reached (e.g. a long and thin ovipositor can oviposit between long and dense hairs). We can divide changes in host use in close, intermediate and distant host changes. For monophagous insects a close change in host use generally involves a plant species within the same plant genus, an intermediate change a different plant genus in the same family and a distant change involves another plant family. As the distance to a different resource increases, utilizing that resource will require an increasing number of simultaneous adaptations to the divergent phytochemistry, different epidermal characteristics and different pattern of interactions with other taxa of the new host [25–29]. Although the odds of a successful distant host change are slim due to the many adaptive requirements, they may be enabled by increased rates of evolutionary change, processes which we are only beginning to understand [30]. According to the "enemy-free space" hypothesis, such changes in the evolutionary rate of change can be linked to the release of predation pressures, or they might be linked to reduced competition for resources, which may counteract the initially lower fitness on a new host [27,31]. Diversification may follow from a distant host shift when this new resource creates access to many resources at intermediate distances. The intermediate resource hypothesis [17] predicts that speciation rates will be highest in situations in which herbivorous insects with limited resource usage (i.e. monophages) are in potential contact with a suite of resources at intermediate resource distances. However, one of the few cases where this was actually tested, a study on the diversification of the subgenus *Drosophila*, showed that speciation rates were not affected by changes in resource use [32].

Although changes in resource use and geographic distribution are two known drivers of diversification in phytophagous insect evolution [5], other candidate drivers should not be discarded beforehand. Increased mortality rates, such as those caused by parasitoid wasps or spiders, can be a strong selection pressure, as was shown by a study on jumping spiders predating on choreutid moths where species that mimicked the appearance and behaviour of the spiders had a higher survival rate [33]. If selective pressures are not continuously present throughout the distribution area of a species, they may lead to disruptive selection. Climatic events are known to have had a strong impact on ecological communities and may have caused bursts of taxonomic diversification in herbivorous insects as well [34]. Furthermore, the factors that drive speciation may influence each other directly or indirectly. Climatic conditions could have created ecological opportunities for host shifts during periods in which different host ranges overlapped. Following from the "enemy-free space" hypothesis, predation pressures may have been a strong selective pressure that favoured individuals that changed their host and were thereby temporarily released from the predation pressure [31,35].

Because many of the aforementioned possible drivers of speciation potentially interacted and may have had a different effect for different groups or at different time periods, disentangling causality from mere correlation among the factors that potentially structure insect-plant networks is a challenging endeavour. This study addresses the phylogenetic relationships within *Ectoedemia* s. str., a subgenus of leafmining moths, and explores the importance of host shifts and allopatry as drivers for speciation. Larvae of leafmining insects are found within plant tissues and are not able to change plant during the larval stage. As a consequence, the host is selected only by the ovipositing female on the basis of visual, olfactory and/or contact chemosensory cues. Her offspring have the choice between surviving on the plant chosen by their mother or perish. There is a significantly higher degree of monophagy in leafminers compared to externally feeding insects, likely because the host not only represents a food resource, but also forms their complete larval habitat. This requires a combination of adapted traits to the various dimensions of the resource [3,8,36]. Ecological factors therefore likely play an important role in speciation in *Ectoedemia*, through changes in host use or one of the dimensions thereof.

With 91 described species, *Ectoedemia* is the largest subgenus within the nominate genus *Ectoedemia* in the leafmining moth family Nepticulidae. All *Ectoedemia* s. str. are leafminers, with one exception of a gall former, whereas some other subgenera display different feeding modes (viz. bark or fruit mining). *Ectoedemia* s. str. is best known from the West Palearctic with 48 described species. From the East Palearctic and Nearctic regions each about 20 species have been recorded. A smaller number of species is known from Africa, Central America and the Oriental region [37–40]. *Ectoedemia* s. str. seems absent from Australia, the Pacific and most of South America, with only a single species known from Belize and Ecuador. In tropical Asia many undescribed species have been collected recently, and will be described in the near future. In North America, only a few more unnamed species are known from collections and recent collecting efforts by the authors and colleagues, suggesting that sampling in that area is now fairly complete. Most *Ectoedemia* s. str. feed on rosid plant orders [41], with the Nearctic as the only exception where some species feed on *Platanus* (Proteales) and *Nyssa* (Cornales). The vast majority of rosid-feeding species is found on just four plant families: Fagaceae, Rosaceae, Salicaceae and Betulaceae. These plant families are ecologically dominant and widespread, particularly in the deciduous forests of the northern hemisphere. *Ectoedemia* s. str. thereby follows the host association patterns of many insect herbivores [3,42]. The resemblance to the gracillariid leafmining genus *Phyllonorycter* and the sawfly subfamily Nematinae (Tenthredinidae, Hymenoptera) is striking, because both these groups also have Fagaceae, Rosaceae, Salicaceae and Betulaceae represented in its top five of most used host plant families [43,44]. Most *Ectoedemia* are monophagous, commonly restricted to a single host genus or even a single host species. Some species within these clades with mostly monophagous species, however, display remarkable diet breadth, e.g. the European *Ectoedemia atricollis*, which not only feeds on a number of rosaceous tree genera, but also on the unrelated genus *Staphylea* (Staphyleaceae) [40].

A phylogenetic study based on morphology has revealed a number of monophyletic species groups in European *Ectoedemia*, of which all species utilise the same host family [40]. Also in North America and the East Palearctic, species have been grouped following morphological similarity, but these groups have been created in regional revisions without evaluating relationships with species from other regions, and have not been analysed following cladistic principles [45,46]. Here, we use a dataset resulting from a largely complete global taxon sampling that includes 92 species from the entire distribution area of the subgenus. Six nuclear and mitochondrial molecular markers are analysed to reconstruct the phylogeny and to test the monophyly of species groups. Thereafter, we superimposed host association and geographic distribution on the obtained tree to evaluate hypotheses on processes of speciation, such as geographic isolation and resource shifts.

## Materials and Methods

### Taxon sampling and marker selection

Taxon sampling of West Palearctic *Ectoedemia* is almost complete, with 44 out of the 47 European species present in our phylogenetic data set, only lacking *Ectoedemia hexapetalae*, *E*. *similigena* and *E*. *aegilopidella*. From the Nearctic 18 species are analysed, including four undescribed ones, only missing two of the named species, viz. *E*. *marmaropa* and *E*. *canadensis* [46,47]. From the East Palearctic we include 12 species and from tropical Asia 26, of which the majority is undescribed [the separation between these biogeographic regions is somewhat arbitrary, here we include all of Taiwan in tropical Asia, although some species are shared with Japan and mainland China]. Furthermore, three species from Africa are included, one unnamed and two named, whereas five named species are known from this area [39,48]. We did not manage to include the only named Neotropical species, *E*. *fuscovittata* [49], the generic assignment of which is somewhat uncertain. Specimens included in this study are all registered on the Barcoding of Life Database (www.boldsystems.org/) under the project “*Ectoedemia* of the World” with detailed information on collecting localities and identification (also available in S1 Table).

The following institutes gave or organised permission for fieldwork and export of specimens in their respective areas: the Mercantour and Alpi Marittime parks in France and Italy (ATBI Mercantour / Alpi Marittime project), the Great Smoky Mountains National Park in the USA, the Institute of Ecology and Biological Resources, Hanoi in Vietnam, the Zoological Institute, Academia Sinica, Beijing in China, the Tropenbos International Indonesia, Balikpapan, Indonesia in Kalimantan, the Korean National Herbarium, Pocheon in South Korea and the National Sun Yat-Sen University, Kaohshiung in Taiwan.

For species that are undescribed we use provisional names, often indicating the distribution area and/or host plant, shown in text between quotation marks; these names also figure in the BOLD database and S1 Table and S5 Table without quotation marks. From the *Ectoedemia* subgenus *Zimmermannia* we include six species as outgroup. In total we used 182 exemplars (174 ingroup and 8 outgroup) in the analyses. Part of this dataset has been analysed in a recent DNA barcoding study [37], which compared the utility of the mitochondrial cytochrome c oxidase subunit I (COI) barcoding gene [50] with the nuclear elongation factor 1 alpha (EF1-alpha) gene for species recognition. Recently obtained material was sequenced at these two loci. These data, in combination with morphology, life history and biogeographic data, were used to assign species boundaries to the undescribed material. The resulting selection contained 92, partly putative, species to be included in the phylogenetic study. Where possible, several representatives per species were included to be able to identify contamination during the molecular work and to detect intraspecific variability. Besides their utility for delimiting species, the COI barcode and EF1-alpha genes are also phylogenetically informative and were therefore included in the present phylogenetic dataset. To resolve the deeper parts of the phylogeny and to increase support in general, four more gene regions were also sequenced, viz. the D2 and D3 domains of the nuclear 28S ribosomal DNA gene, part of the nuclear isocitrate dehydrogenase (IDH) gene, the nuclear histone 3 gene and part of the mitochondrial cytochrome c oxidase II (COII) gene.

### DNA extraction

DNA extractions were performed with a Qiagen DNeasy blood & tissue kit or with a Macherey Nagel magnetic bead tissue kit on an automated KingFisher flex system. Different types of tissue were used for extraction, depending on the abundance and significance of the specimens available. Hind legs were carefully removed from adults and cut into pieces with a scalpel. Non-destructive abdomen extractions were used so that genitalia preparations could be combined with DNA extraction [51]. Larvae were ground with a disposable pestle, or incisions were made in the cuticle to perform a non-destructive extraction of tissue material, after which the larval pelt was embedded in euparal on a microscopic slide.

### PCR

Details on primers, including references, are listed in S2 Table. We sequenced the mtDNA cytochrome c oxidase I (COI) barcode region, which was 658 bp in length, sometimes in two parts if the material was degraded. Primer sets LepF1 and LCO1490 and LepR1 and HCO2198 attach to the same region and were often combined in a single PCR to obtain a higher DNA yield. A fragment of nuclear elongation factor 1-α (EF1-α) was 482 bp in length, and was sometimes also amplified in two parts. The D2 and D3 regions of the 28S ribosomal DNA gene had an aligned length of 873 bp. For the cytochrome oxidase II (COII) fragment, primer “eva” attaches to the tRNA-Lysine region at the 3' end of COII and this fragment was pruned for a stable aligned COII length of 638 bp; histone 3 sequences were 328 bp in length and isocitrate dehydrogenase (IDH) sequences 723 bp. A PCR cycle consisted of 3 minutes initial denaturation at 94°C, 15 seconds denaturation at 94°C, 30 seconds at the optimal annealing temperature, and 40 seconds extension at 57°C. A final extension at 72°C for 5 minutes completed the reactions. The optimized annealing temperature for COI and COII was 50°C, for histone 3 and IDH 55°C, and for EF1-alpha and 28S rDNA 57°C. Universal tails were attached to the primers to facilitate higher throughput and to increase yield [52,53]. PCR reactions were performed in volumes of 25 μl. Purification and bidirectional sequencing were outsourced to Macrogen Europe or Baseclear Leiden. Sequencher 4.2 (Gene Codes corporation) or Geneious R6 (www.geneious.com/) software was used to align the forward and reverse sequences, to manually check for ambiguities in the chromatograms and to create contigs. The COI, IDH, histone 3 and EF1-alpha alignments contained no gaps and were aligned by eye. COII contained a 3-bp insertion in *Ectoedemia quadrinotata* only. We used secondary structure to align the 28S rRNA sequences, despite the fact that within *Ectoedemia* there are only few ambiguous areas, making the alignment straightforward. Sequences were stored using the open-source package VoSeq 1.6.0 [54].

### Phylogenetic analyses

Neighbor-joining trees were created using PAUP 4b10 [55] for each gene to assess congruence between markers. Alignments of different markers were then concatenated using VoSeq 1.6.0 and manually checked in Geneious R6, resulting in the preliminary dataset 'EctZimm2' with 187 exemplars representing 98 species. The most appropriate model for the phylogenetic analyses was determined with the online Findmodel service [56]. Single-gene as well as concatenated datasets were tested, and for all datasets the GTR+Gamma model was calculated to be most likely and used in subsequent analyses. Bayesian analyses were performed with MrBayes v3.2.1 [57,58] on the Oslo Bioportal [59] and Cipres Portal [60]. Maximum likelihood analyses were done using the PhyML 2.2.0 plugin in Geneious R6 and RaxML HPC2 7.7.4 and Garli 1.0 on the Cipres portal. RaxML analyses were done both without partitions and with mtDNA, nuDNA and rDNA sequences separated in three partitions; these all resulted in highly similar tree topologies and branch support values. The resulting RaxML bootstrap trees from the initial analysis with all 187 exemplars were analysed with Roguenarok [61] to search for exemplars negatively influencing support values. The Roguenarok majority-rule consensus (MR) and extended majority rule consensus (MRE) algorithms flagged *Ectoedemia suberis* (RMNH.INS.12705), *E*. *“Namibia”* (RMNH.INS.23989), *E*. *aegilopidella* (RMNH.INS.23875) and both *E*. *picturata* specimens (RMNH.INS.23888 & RMNH.INS.23891), all exemplars that were missing sequence information for several markers. These taxa were subsequently removed in different steps, finally resulting in the dataset 'EctZimm5' with 182 exemplars representing 92 species. The analyses were then repeated on this culled dataset, after which there were no more exemplars flagged by Roguenarok. The increase in support values for key clades following the removal of "rogue" examplars is shown in S3 Table. Garli maximum likelihood bootstrap (BS) and MrBayes Bayesian posterior probability (PP) support values were plotted on the respective branches of the PhyML best tree. We considered bootstrap values between 60 and 80 as moderate support, values above 80 as high support. For the posterior probabilities, we considered values of 0.85 to 0.95 as moderate support, and values above 0.95 as high support. We indicated higher clades with abbreviations of the species groups found within these clades, for example SUPO for the combination of the *Ectoedemia subbimaculella* and *Ectoedemia populella* groups.

### Character mapping

Mesquite version 2.75 (build 566) was used to map characters on the phylogenetic tree. The complete PhyML best tree with 182 exemplars was pruned so that a single terminal branch per species remained. Host plant family and geographic distribution were then plotted on the tree using the Trace Character History option under the Maximum Parsimony reconstruction with unordered states. This criterion calculates the fewest state changes required without assuming a mode of evolution or inferring information from the branch lengths. The results therefore often include equally likely states for many of the basal nodes, but indicate the strongest patterns in the data. The two resulting trees were arbitrarily ultrametricized and mirrored to allow for visual recognition of patterns between biogeography and host usage. Host plant associations at plant family level are mapped onto the phylogeny to identify distant changes in host use. The feeding mode, leafmining, is the same throughout the subgenus, with only one exception: the petiole galling *Ectoedemia populella*. Feeding mode is therefore not further analysed. Some species are specialized on a single host plant species, but most utilize several species within the same genus. A small number is oligophagous within a single plant family or disjunct oligophagous. In cases where more than one host plant species is used there often is a preference hierarchy of the ovipositing females that may vary throughout their distribution [62]. In this study we focus on host changes at the plant family level and evaluate how these correlate with shifts between major biogeographic regions. In cases in which a species can be found on multiple host families, only the dominant host has been indicated. To be able to identify shifts in distribution area, i.e. allopatry, throughout the phylogeny, the respective biogeographic region(s) of species has been mapped as well. We followed the general definitions of biogeographic regions [63], except that the Palearctic has further been divided into a West and East part with the “Turgai Strait”, roughly following the 64–65 meridian as the divide [38], to allow for a more precise delineation of species’ distributions. It should be noted though that this is still a rough estimate of species distributions, the actual distribution of most species only includes a smaller section of a biogeographic region and shifts within a single biogeographic region remain undetected.

## Results

### A global scale phylogeny of the subgenus *Ectoedemia*

**Fig 1:**
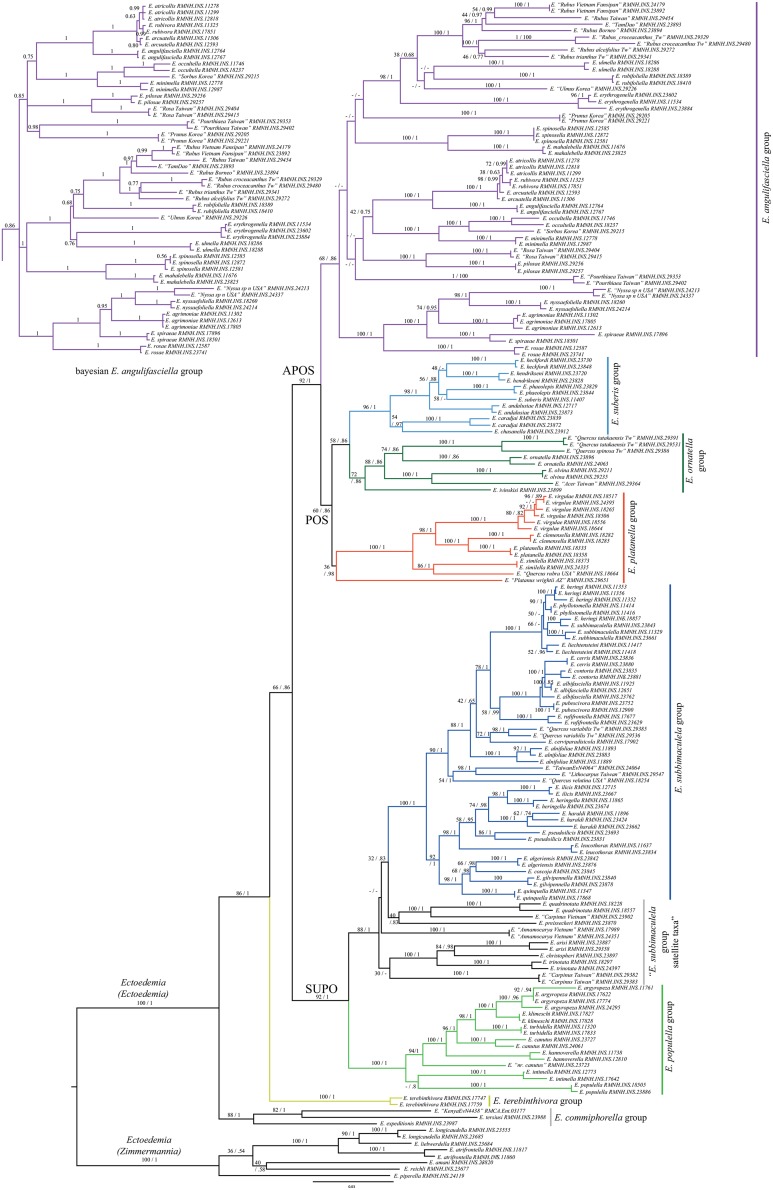
Phylogeny of *Ectoedemia* s. str.

The complete dataset contains 3692 bp from six genetic markers for 174 ingroup and eight outgroup exemplars, representing a largely complete global taxon sampling with 92 ingroup species. See S4 Table for Genbank accession numbers. Data from at least three DNA markers, encompassing a minimum of 1472 bp, was available for each taxon. The phylogeny based on the 3692 bp of the six markers combined is generally well resolved and supported (Fig. 1). The Maximum Likelihood and Bayesian analyses provided similar topologies, allowing the support values to be plotted on the respective branches of the best Maximum Likelihood tree. The delimitation for species groups that we propose is indicated with coloured clades in the phylogeny (Fig. 1). The resulting new classification with all described and undescribed, but included in the phylogeny, *Ectoedemia* species is provided in S5 Table and is outlined as following:

Genus *Ectoedemia* Busck, 1907

Subgenus *Zimmermannia* Hering, 1940

Subgenus *Ectoedemia* s. s.

*E*. *commiphorella* group

*E*. *terebinthivora* group

SUPO—clade

*E*. *populella* group

*E*. *subbimaculella* group—satellite taxa

  [includes former *E*. *preisseckeri* group]

*E*. *subbimaculella* group

APOS—clade

 POS-clade

*E*. *platanella* group

*E*. *ornatella* group

*E*. *suberis* group

*E*. *angulifasciella* group

  [includes former *E*. *rubifoliella* and *occultella* groups]

PhyML best tree resulting from an analysis of 182 exemplars and six DNA markers, totalling 3692 bp. The outgroup contains six taxa of the subgenus *Ectoedemia* (*Zimmermannia*). In total 92 species of *Ectoedemia* s. str. are included. Species groups are colour coded and the support values of the Maximum Likelihood and Bayesian analyses are plotted on the branches. Because the topology of the *E*. *angulifasciella* group differed substantially, both the ML and Bayesian results are shown (see also S3 Table).

Only in the clade with the *angulifasciella* group there are substantial differences between the Bayesian and Maximum Likelihood analyses (Fig. 1). This is mainly due to the different position of *Ectoedemia "Prunus_Korea"*, which is sister to *E*. *"Pourthiaea_Taiwan"* in the Bayesian analysis but found basal to a clade with predominantly *Rubus*-feeding species in the Maximum Likelihood analysis, resulting in a different basal structuring of the *angulifasciella* group. The group as a whole is recovered as monophyletic in both phylogenetic analyses be it with medium statistical support.

Not all species could be assigned to a monophyletic species group. The "satellite taxa" basal to the *subbimaculella* group are all found in the same region of the molecular phylogeny, but cannot be placed in well-supported monophyletic species groups that are congruent with those based on morphology. Among these taxa are the former monotypic *E*. *preisseckeri* group, as well as the species *Ectoedemia christopheri* and *E*. *trinotata*, which have previously been regarded as part of the *populella* group on the basis of their genitalia [45,46]. Besides the species groups, several other higher clades receive high support. Disregarding the basal section of the tree with the *E*. *commiphorella* and *E*. *terebinthivora* groups, there is a split into two large clades, named here as SUPO and APOS. The *subbimaculella* group with its basal satellite taxa and the *populella* group are placed together in the SUPO clade (BS = 92, PP = 1). The second clade, APOS, is recovered with high support (BS 92, PP 1) and contains the *angulifasciella* group as well as a less well-supported clade named POS (BS 60, PP 0.86), containing the *platanella*, *ornatella* and *suberis* groups. Below we treat each species group in detail, the detailed new classification is presented in S5 Table.

### The *E*. *commiphorella* and *E*. *terebinthivora* groups

The African taxa form the most basal split-off from the outgroup. Two of the three taxa included in the molecular phylogeny have been described, but they have previously not been attributed to any species group. Although the host plants of the African species that we sequenced are unknown, the hosts for two described closely related *Ectoedemia* are *Commiphora* species (Burseraceae) [64,65], and it is not unlikely that other African species also feed on this widespread and diverse plant genus. We here erect the *Ectoedemia commiphorella* group for the five named (including *E*. *commiphorella*) and the two unnamed African *Ectoedemia* species, which are all morphologically similar to each other [48,64,65], and constitute a monophyletic group in the molecular phylogeny. The *E*. *terebinthivora* group contains only *E*. *terebinthivora* and represents a split-off between the *E*. *commiphorella* group and the rest of the ingroup. The species is found in the eastern Mediterranean area and is unique in that it feeds on Anacardiaceae (it is monophagous on *Pistacia terebinthus*). The unique position as a stand-alone species in the molecular analysis is concordant with its position in the morphological tree [40].

### The *E*. *subbimaculella* group

Within the SUPO clade, the *Ectoedemia subbimaculella* group is recovered with high support. All species in this group feed on oaks and relatives (Fagaceae, primarily the genus *Quercus*). The group is well defined by morphological characters and is monophyletic in the molecular phylogeny with high support. Comparing the molecular results with published results on a subset of the European species that was previously assigned to this group based on morphology [40], displays remarkable similarities. In both phylogenies there is a clade around *E*. *quinquella* (best recognised by the characteristic forewing colour pattern and presence of a hair pencil at the base of the male hindwing), a southern European clade composed of species that feed on evergreen oaks (*E*. *leucothorax*, *E*. *pseudoilicis*, *E*. *haraldi*, *E*. *ilicis* and *E*. *heringella*) and a clade comprising the *subbimaculella* complex, *E*. *rufifrontella* and the *albifasciella* complex (sensu [40]). The latter clade is expanded in the molecular phylogeny and has at its basis *E*. *alnifoliae*, several Asian species and the single North American representative of the *subbimaculella* group, a single larva found on *Quercus velutina* in the Great Smoky Mountains (USA: North Carolina).

### The *E*. *populella* group

Also part of the SUPO clade, the *populella* group is recovered with high support in all analyses. It consists of a variety of Palearctic and Nearctic species, including the type species of the genus, the Nearctic *Ectoedemia populella*. The group is well-defined morphologically [40], amongst others by the presence of two pairs of strongly developed carinae in the male genitalia. The group contains all Salicaceae-feeding *Ectoedemia*: *Ectoedemia intimella* feeds on *Salix* spp., whereas all other species feed on *Populus* spp. All first instars of these species start feeding in the petiole or midrib, and only later produce a leafmine, except *E*. *populella* which makes a gall in the petiole. The undescribed *E*. *"nr canutus"* also belongs to this group.

### The *E*. *angulifasciella* group

The *angulifasciella* group is the largest species group of the APOS clade and is recovered with medium statistical support (BS 68, PP 0.86). When the group was erected based on morphology there was some uncertainty about its monophyly, as there were no clear common apomorphies to distinguish the group as a whole [40]. Nonetheless, the delimitation based on morphology and DNA is congruent and furthermore, it comprises all Rosaceae-feeding *Ectoedemia* species. Only a few species in this group feed on other plant families, viz. Betulaceae (the former *occultella* group), Ulmaceae (two species) and Cornaceae (two North American *Nyssa*-feeding species). The Maximum Likelihood and Bayesian analyses differed in the structuring of the *angulifasciella* group, due to a different position of the undescribed *E*. *"Prunus_Korea"*; both alternatives are shown in Fig. 1. Disregarding the position of *E*. *"Prunus_Korea"*, the *angulifasciella* group is divided into three larger subclades in both the Bayesian and Maximum Likelihood tree. The first group consists of the *E*. *rubivora* cryptic species complex + *E*. *angulifasciella* and *E*. *occultella*, *E*. *minimella* and E. "*Sorbus_Korea*", together with four East Palearctic species (PP 0.85). The second large clade (PP 1) contains almost all *Rubus*-feeding *Ectoedemia* and two *Ulmus*-feeding species, with the West Palearctic species pair *E*. *spinosella* and *E*. *mahalebella* as sister clade. The *Rubus*-feeding taxa from Southeast and East Asia show a remarkable molecular diversity: specimens that look morphologically almost the same seem to form a complex of sibling species, with at least several clearly differentiated taxa in Taiwan and Vietnam. The third clade (PP 1) groups the North American *Nyssa*-feeding clade with three West Palearctic Rosaceae feeders.

### The POS clade: *E*. *platanella*, *E*. *ornatella* and *E*. *suberis* groups

The POS clade spans three species groups that are each found in a different biogeographic region. The *E*. *platanella* group is Nearctic, the *ornatella* group is East Palearctic/Oriental and the *suberis* group is West Palearctic. The *E*. *platanella* group is morphologically characterised by very similar male genitalia with multi-branched setae on the inner surface of the valves as a unique apomorphy [46]. It was initially only known from eastern North America. In the molecular phylogeny the group is recovered with high support, but support is much lower if the undescribed western North American species, collected as larva from *Platanus wrightii*, is included (RMNH.INS.29651). The Arizonan larvae of this host may belong to the same species that has been reared from *Platanus racemosa* in California; we have tentatively included it in the *platanella* group. Although specimens found on *Carya* were initially considered to be a different species, genitalia and DNA proved them to be conspecific with *Ectoedemia virgulae*. This means that *E*. *virgulae* is disjunct oligophagous, with larvae commonly found feeding on *Corylus* (Betulaceae) as well as *Carya* (Juglandaceae). In fact, this entire species group is characterised by distant host plant shifts: three species feed on Platanaceae, one on Fagaceae and one on Betulaceae and Juglandaceae. In contrast to the distant host shifts in the *platanella* group, all species in the *ornatella* and *suberis* groups feed on plant species within a single host genus (*Quercus* spp.) (see [37,38], with as only exceptions *E*. *olvina* and its undescribed sister species *E*. *"Acer_Taiwan"* which both feed on *Acer* (Sapindaceae). The *ornatella* and *suberis* group delimitations in the molecular phylogeny are congruent with the delimitation based on morphology.

### Host plant associations

**Fig 2:**
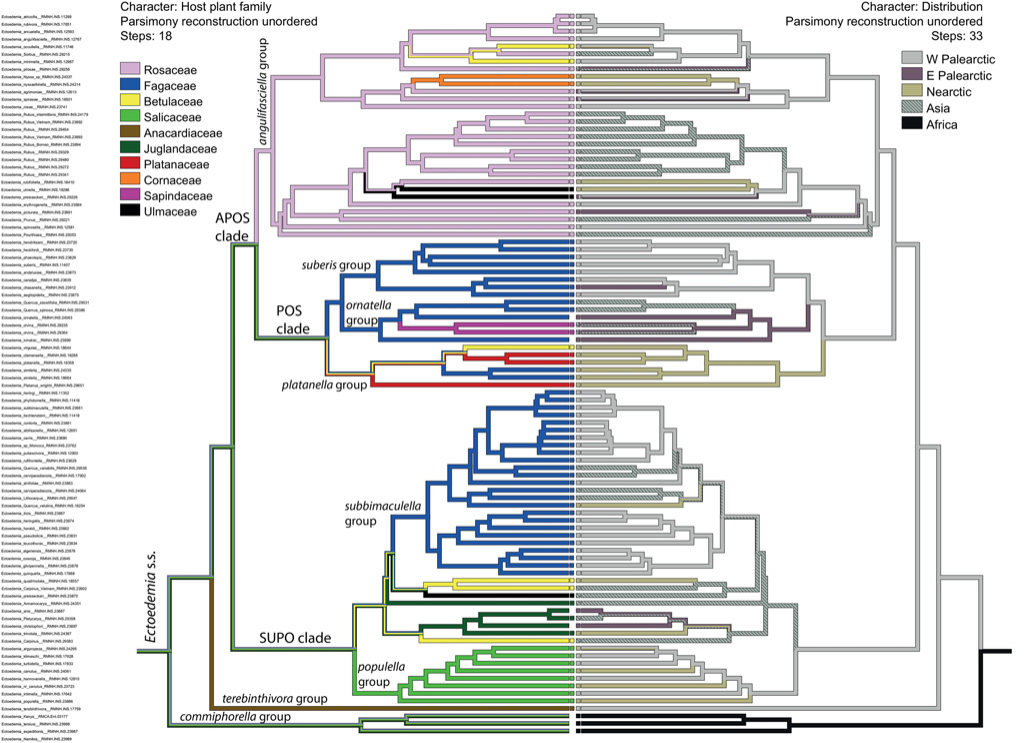
Ancestral state reconstruction of biogeography and host family.

The Maximum Parsimony reconstruction of biogeographic ancestral states (left tree) juxtaposed to those of host family (right tree) is shown in Fig. 2. Taxa with squares indicate that both host and biogeography are known, for those that do not have squares for the host, this information is reconstructed. Eighteen changes are required to explain the observed pattern of host use at family level. For all species groups except the *platanella* group, the ancestral host can unambiguously be assigned. The ancestral host of the *angulifasciella* group most likely fed on Rosaceae, and represents the only shift to Rosaceae in the subgenus *Ectoedemia*. Fagaceae are the most likely ancestral host for three species groups: the *suberis* group, the *ornatella* group and the *subbimaculella* group. Similar to Rosaceae, Salicaceae have only been colonised once, by the ancestor of the *populella* group. The ancestral host for the *platanella* group remains ambiguous and may have belonged to the Platanaceae, Fagaceae or Betulaceae. More basal in the tree, however, the ancestral host remains ambiguous. The ancestors of the APOS, POS and SUPO clades fed equally parsimoniously on Rosaceae, Fagaceae, Betulaceae, Salicaceae, Anacardiaceae, Juglandaceae and Platanaceae. The same seven plant families are the equally parsimonious ancestral hosts further up the tree towards the outgroup. The hosts of the outgroup taxa have purposely not been indicated to avoid bias in the analysis. The host plant association results corroborate earlier morphological studies that showed that most species groups are found on one host plant family only.

Ancestral state reconstruction of biogeography (right) and host plant family (left) in two mirrored trees with topology from the Bayesian analysis. Squares in the centre indicate for a taxon whether the information was known (square present) or reconstructed (square absent). Only the dominant family is indicated in case a species uses more than one host family. Multiple colours on a branch indicate ambiguous reconstructed states, except for the terminal branches where they indicate multiple states for that taxon.

### Biogeography

There are more changes in biogeography throughout the phylogeny than there are changes in host plant family. A minimum of 33 changes is required to explain the observed pattern in biogeography, whereas only 18 host family changes are needed to explain the host plant family pattern. These shifts in biogeography are not evenly distributed over the tree, instead they are most common in the *Ectoedemia angulifasciella* group, where a biogeographic shift is involved for 43% of the speciation events. Biogeographic shifts are less common in the SUPO clade in which there is a shift for 31% of the speciation events and fewest in the POS clade with 25%. Just four species are known to occur in more than one biogeographic region: *E*. *argyropeza*, *E*. *intimella*, *E*. *occultella* and *E*. *spiraeae*. Of these, DNA barcoding and allozyme studies have shown that *E*. *argyropeza* in the Nearctic reflects a recent European introduction [37,66]. However, a large pairwise intraspecific distance of 6.85% in DNA barcodes of *E*. *spiraeae* and a similarly large distance of 6.5% in DNA barcodes of *E*. *intimella* [37] suggests that these species have a wide distribution throughout the Palearctic, although we cannot entirely rule out the possibility that different species are involved, as long as specimens in the area between Europe and East Asia not have been genotyped. A 3.0% COI barcode pairwise distance between *E*. *occultella* specimens from the Nearctic and Europe suggests that also this species exhibits an old Holarctic distribution rather than a recent introduction into the Nearctic. Nonetheless, such vast distribution areas are exceptional, the great majority of species are restricted to a single biogeographic region. The biogeographic origin of the *angulifasciella*, *suberis*, *populella* and *terebinthivora* species groups is most likely the West Palearctic. The *subbimaculella* group ancestor could have lived equally parsimoniously in the West Palearctic or Asia. The *ornatella* group origin is reconstructed to be Asian and the *platanella* group of a Nearctic origin. A West Palearctic origin seems most likely for all higher clades, including the POS, the APOS, the SUPO and the SUPO + *terebinthivora* clade. Only the clade with the *commiphorella* group, with its African origin, causes the parsimony reconstruction to place the origin of the whole subgenus in Africa.

## Discussion

### Molecules and morphology

Our molecular phylogeny of 92 species represents the most complete phylogeny for *Ectoedemia* to date. Currently, 91 species are described, of which 65 are represented in this phylogeny, the remaining 27 species that we included are putative species. The six molecular markers used in this study were able to resolve most clades with good statistical support. Only the section of the tree with the "*subbimaculella* group satellite taxa" remains unresolved and requires more taxa and/or genes to get fully resolved. The phylogeny allowed us to evaluate published delimitations of species groups proposed on the basis of morphology [38,40,45,47]. All monophyletic species groups that are proposed here already had been named previously, with the exception of the newly erected *E*. *commiphorella* group. However, most groups include more species than originally conceived, some of which were originally unplaced, and some were placed in other species groups that were not recovered by our phylogenetic analyses. Overall, the molecular results corroborate the existing phylogenetic—morphology based—relations of the species groups.

The relations among species groups long remained unclear in morphological studies as a result of the many homoplasious characters [40]. We have now been able to resolve these relationships and we assign three overarching clades, named APOS, POS and SUPO, to indicate basal splits in the evolution of the subgenus. The APOS and SUPO clades each contain about half the total number of species. Two small clades are even more basal: the *commiphorella* group containing the African taxa and the monotypic *E*. *terebinthivora* group. The species-rich APOS clade contains the *angulifasciella*, *platanella*, *ornatella* and *suberis* groups, and within this clade the *angulifasciella* group splits off, leaving the POS clade with the *platanella*, *ornatella* and *suberis* species groups. The second large clade, SUPO, contains two species rich groups, the *subbimaculella* and *populella* species groups, as well as several unplaced taxa that fall between these two groups. The full new classification is provided in S5 Table.

The generally good resolution and support values allowed us to map two characters that are likely drivers of speciation, viz. host plant family shifts and biogeography, onto the obtained phylogeny. The parsimony reconstruction that we used is sensitive to changes in taxon sampling, because a single added taxon with a different host family may have a large effect on the amount of changes required for a large group of taxa. However, as taxon sampling is fairly complete, we can reliably use our data to explore the role of host plant family shifts and biogeography in the evolution of *Ectoedemia*.

### Co-evolution?

Although co-evolution is not strictly tested in our analyses, it is evident that the shifts to different plant families never follow host phylogeny [41]. At most it can be said that the four plant families that are consumed by three quarters of the *Ectoedemia* s. str. species fall within the APG fabid (= rosid I) clade, but the remaining species use plants in the order Sapindales [malvid (= rosid II) clade], in the more distantly related order Proteales (basal eudicots) or in the order Cornales (basal in the asterid clade). This indicates that co-speciation or parallel cladogenesis at this phylogenetic level, similar to many groups of herbivorous insects [16], is highly unlikely. Instead, the pattern of changes in host plant family use that we revealed suggests repeated colonisation of different host plant families, congruent with a 'resource archipelago' scenario, and some of these colonisations have been followed by substantial diversification, which may be congruent with the 'intermediate resource hypothesis'.

### Diversification following a host plant family shift

Species group boundaries, initially independently defined by morphological characters, commonly coincide with shifts to a different host plant family, indicating that the host plant family shift was accompanied by morphological change. Host plant family conservatism within species-rich clades like we find in *Ectoedemia* is commonly found among insect herbivores [14,67,68], and fits the hypothesis that such a change involves a complex of simultaneous adaptions and is therefore phylogenetically constrained. However, once conceived, it may open a world of resources at intermediate distances. The four host plant families that include the largest radiations of *Ectoedemia*, host 78% of the *Ectoedemia* s. str. species that we included in our phylogeny: Fagaceae (40%), Rosaceae (24%), Salicaceae (8%) and Betulaceae (6%). Of these, Rosaceae and Salicaceae are reconstructed to have been colonised once, Fagaceae two or three times and Betulaceae three to five times. These four plant families all comprise a large diversity of, often ecologically dominant, plant species in the Holarctic that may constitute resources at intermediate distances from the initial host change towards the family. The plant families that do not host substantial *Ectoedemia* radiations are, at least in the Holarctic, relatively species-poor (viz. Anacardiaceae, Juglandaceae, Platanaceae, Cornaceae, Sapindaceae and Ulmaceae). Finally, it should also be noted that the main host families of *Ectoedemia* are also host to many other leaf-mining insects [3,44], suggesting that these plant families in general have one or more common denominators that have made them suitable for colonisation and speciation. The balance between the odds for colonisation and the possibilities for subsequent diversification are likely not equal between the different host plant families. For example for Betulaceae, the abundance of species utilizing this host plant family can most easily be explained by several independent shifts to this family with little subsequent diversification. There are also indications from other groups that there is a large chance to colonise Betulaceae; a combination of Rosaceae and Betulaceae as hosts as seen in the *E*. *angulifasciella* group is known for various leafminer groups. Some share these two host families even within one species [e.g. *Lyonetia clerkella* (Lyonetiidae) and *Phyllonorycter corylifoliella* (Gracillariidae); [69,70].

### Allopatric speciation

The borders of the biogeographic regions erected by Wallace [63] that we use here are major barriers to gene flow for most animals. The regions are mostly defined by different climatic conditions in combination with geographic distance. In *Ectoedemia* we observe that the whole subgenus is mostly restricted to the biogeographic regions of the temperate northern hemisphere, and that the vast majority of species is restricted to a single biogeographic region. Restricted intraspecific gene flow over large geographic distances is further corroborated by the COI barcode distances [37], indicating genetic isolation between conspecific specimens from different regions. However, for many species the faunistic knowledge is currently too scarce to know their actual distribution. For those species where we do have rather complete information, the distribution rarely exactly coincides with the boundaries of a biogeographic region but includes only a smaller area. As a consequence, we cannot accurately estimate for the whole subgenus where species distributions may overlap or not and the 33 reconstructed shifts between biogeographic regions provide only a minimum estimate of the speciation events of which we are fairly certain that they involved a geographic component. Focussing on the larger patterns however, it can generally be said that within and between species groups, shifts between biogeographic regions are more common than shifts to another host plant family. To further unravel the extent of the effect of geographic isolation as a driver of speciation, it will be crucial to examine the population genetic structure of species with wide distributions for isolation by distance patterns.

### Different drivers for different clades

Although we find general patterns with regards to host family shifts and biogeography, none appear universally applicable for the whole subgenus. An exception to the general pattern that host plant family is a more conservative character within species groups than biogeographic occurrence is the POS clade, which includes the three smallest polytypic groups. The *E*. *platanella* group is exclusively Nearctic, the other two groups have representatives either throughout the Palearctic (*suberis* group) or Asia and the East Palearctic (*ornatella* group). Fagaceae host *Ectoedemia* in all biogeographic regions, but the *Ectoedemia* s. str. fauna is especially rich in southern Europe with many representatives from both the *subbimaculella* and *suberis* groups. The lack of a diverse *Ectoedemia* fauna feeding on Fagaceae in the Nearctic is surprising, considering the large radiation of Nearctic *Quercus* (e.g. [71]) as potential resources at intermediate distances. A very different dimension of speciation might be evident in the *E*. *populella* group. *Ectoedemia populella* is the only *Ectoedemia* s. str. with a clearly different feeding mode, petiole galling, and the biology of the entire group is somewhat divergent. The egg is laid on the petiole or midrib of the leaf where the mine starts and only later enters the leaf, instead of directly starting in the leaf [40]. The leafmining larvae of species in the *populella* group are able to withdraw in the petiole and this adaptation may have been a factor in the emergence and diversification of this species group, possibly strengthened through the combination with an initial adaptation to a different phytochemistry in allopatry. Clearly, neither shifts between biogeographic regions or host plant families alone can explain speciation in *Ectoedemia*. The two factors have possibly acted simultaneously, through differences in the availability of hosts in different parts of the world at different times, and their relative importance for speciation therefore varied for different clades.

## Conclusions

1. Our molecular results comprise the most complete overview of the phylogenetics of the subgenus *Ectoedemia* and provide a solid base for the erection of eight monophyletic species groups: *E*. *commiphorella* group, *E*. *terebinthivora* group, *E*. *populella* group, *E*. *subbimaculella* group, *E*. *platanella* group, *E*. *ornatella* group, *E*. *suberis* group and *E*. *angulifasciella* group.

2. Our character state mapping analyses provide a coarse view of the role of geographic isolation and host plant shifts during speciation events in *Ectoedemia* and reveals some general patterns as well as exceptions to this pattern. Species groups are generally conserved in their host plant family, more so than their biogeographic regions, although in the POS clade this pattern is reversed.

3. Distant host shifts did not trace plant evolution, and diversification has not always followed from a distant host shift. Diversification has followed from shifts to Rosaceae, Salicaceae and Fagaceae, but not from shifts to Betulaceae, Platanaceae, Anacardiaceae, Juglandaceae, Cornaceae, Sapindaceae and Ulmaceae.

4. Neither biogeography nor host plant family alone can explain the speciation patterns we find in *Ectoedemia* s. str., instead, a combination of these and other factors has likely been important, and has likely been differently for different clades.

## Supporting Information

S1 Table Collecting, identification and voucher information of all the material used in the study. (XLS)

S2 Table Primer names, forward and reverse primer sequences and references. (DOCX)

S3 Table Support increases for different datasets during Garli runs. (DOCX)

S4 Table Identifiers, taxonomy and Genbank accession numbers of the material used in this study. (DOCX)

S5 Table New Classification of *Ectoedemia* (*Zimmermannia*) and *Ectoedemia* s. str., including unnamed species. (XLS)
