# Supplementary material for: A Global Phylogeny of Leafmining Ectoedemia Moths (Lepidoptera: Nepticulidae): Exploring Host Plant Family Shifts and Allopatry as Drivers of Speciation
Source: PLoS One. 2015 Mar 18;10(3):e0119586. doi: 10.1371/journal.pone.0119586 (PMC4365004; doi:10.1371/journal.pone.0119586)
Supplement: S2 Table — (DOCX) [file pone.0119586.s002.docx]

**Supporting Information 2 to:**

**A global phylogeny of leafmining *Ectoedemia* moths (Lepidoptera: Nepticulidae): exploring host plant family shifts and allopatry as drivers of speciation.**

Camiel Doorenweerd, Erik J. van Nieukerken & Steph B. J. Menken

PLoS one

**SI2. Primer names, forward and reverse primer sequences and references.**

| Primer | Reference | Direction | Target | Sequence 5' - 3' |
| --- | --- | --- | --- | --- |
| M13F | [1] | Forward | universal tail | TGTAAAACGACGGCCAGT |
| M13R | [1] | Reverse | universal tail | CAGGAAACAGCTATGAC |
| LepF1 | [2] | Forward | COI-5p | ATTCAACCAATCATAAAGATATTGG |
| LepR1 | [2] | Reverse | COI-5p | TAAACTTCTGGATGTCCAAAAAATCA |
| LCO1490 | [3] | Forward | COI-5p | GGTCAACAAATCATAAAGATATTGG |
| HCO2198 | [3] | Reverse | COI-5p | TAAACTTCAGGGTGACCAAAAAATCA |
| mLepF1 | [4] | Forward | COI-5p | GCTTTCCCACGAATAAATAATA |
| mLepR1 | [4] | Reverse | COI-5p | CCTGTTCCAGCTCCATTTTC |
| F | [5] | Forward | COII | GGAGCATCTCCTTTAATAGAACA |
| eva | [6] | Reverse | COII | GAGACCATTACTTGCTTTCGATCATCT |
| S3660F | [7] | Forward | 28S | GAGAGTTMAASAGTACGTGAAAC |
| A335R | [8] | Reverse | 28S | TCGGARGGAACCAGCTACTA |
| EF-NepF | [9][10] | Forward | EF1-alpha | GCCCCCGGACACAGAGATTTCA |
| EF-NepR | [9] | Reverse | EF1-alpha | CACGACCTACTGGCACTGTTCC |
| EF-mNepF | [9] | Forward | EF1-alpha | CCCAGATTYGARGAAATYAAR |
| EF-mNepR | [9] | Reverse | EF1-alpha | GCAACDGCAGCTGGRTTRTA |
| IDHdeg27F | [10] | Forward | IDH | GGWGAYGARATGACNAGRATHATHTGG |
| IDH-NepR | this publication | Reverse | IDH | TTRCAGGCCCANACGAANCCNCCYT |
| HexAF | [11] | Forward | Histon3 | ATGGCTCGTACCAAGCAGACGGC |
| HexAR | [11] | Reverse | Histon3 | ATATCCTTGGGCATGATGGTGAC |

**References to table SI2**

1. Messing J (1983) New M 13 vectors for cloning. Methods in Enzymology.

2. Hebert PDN, Cywinska A, Ball SL, DeWaard JR (2003) Biological identifications through DNA barcodes. Proceedings of the Royal Society of London Series B-Biological Sciences 270: 313-321.

3. Folmer O, Black M, Hoeh W, Lutz R, Vrijenhoek R (1994) DNA primers for amplification of mitochondrial Cytochrome C Oxidase subunit I from diverse metazoan invertebrates. Mol Mar Biol Biotechnol 3: 294-299.

4. Hajibabaei M, Janzen DH, Burns JM, Hallwachs W, Hebert PDN (2006) DNA barcodes distinguish species of tropical Lepidoptera. Proceedings of the National Academy of Sciences of the United States of America 103: 968-971.

5. Sperling FA, Landry J-F, Hickey DA (1995) DNA-based identification of introduced ermine moth species in North America (Lepidoptera: Yponomeutidae). Annals of the Entomological Society of America 88: 155-162.

6. Caterino MS, Sperling FA (1999) Papilio phylogeny based on mitochondrial Cytochrome Oxidase I and II genes. Molecular Phylogenetics and Evolution 11: 122-137.

7. Morse GE, Normark BB (2006) A molecular phylogenetic study of armoured scale insects (Hemiptera: Diaspididae). Systematic Entomology 31: 338-349.

8. Whiting MF, Carpenter JC, Wheeler QD, Wheeler WC (1997) The Strepsiptera problem: phylogeny of the holometabolous insect orders inferred from 18S and 28S ribosomal DNA sequences and morphology. Systematic Biology 46: 1-68.

9. van Nieukerken EJ, Doorenweerd C, Stokvis FR, Groenenberg DSJ (2012) DNA barcoding of the leaf-mining moth subgenus Ectoedemia s. str. (Lepidoptera: Nepticulidae) with COI and EF1-α: two are better than one in recognising cryptic species. Contributions to Zoology 81: 1-24.

10. Wahlberg N, Wheat CW (2008) Genomic outposts serve the phylogenomic pioneers: Designing novel nuclear markers for genomic DNA extractions of lepidoptera. Systematic Biology 57: 231-242.

11. Ogden TH, Whiting MF (2003) The problem with "the Paleoptera Problem:" - sense and sensitivity. Cladistics 19: 432-442.
