# Supplementary material for: A Global Phylogeny of Leafmining Ectoedemia Moths (Lepidoptera: Nepticulidae): Exploring Host Plant Family Shifts and Allopatry as Drivers of Speciation
Source: PLoS One. 2015 Mar 18;10(3):e0119586. doi: 10.1371/journal.pone.0119586 (PMC4365004; doi:10.1371/journal.pone.0119586)
Supplement: S3 Table — (DOCX) [file pone.0119586.s003.docx]

**Supporting Information 3 to:**

**A global phylogeny of leafmining *Ectoedemia* moths (Lepidoptera: Nepticulidae): exploring host plant family shifts and allopatry as drivers of speciation.**

Camiel Doorenweerd, Erik J. van Nieukerken & Steph B. J. Menken

PLoS one

**SI3: Supporting information table 3. Support increases for different datasets during Garli runs after removing ‘rogue’ taxa.**

| Clade (figure I) | Dataset  EctZimm2 | Dataset  EctZimm5 |
| --- | --- | --- |
| APOS clade | 24 | 92 |
| *E. angulifasciella* group | 14 | 68 |
| POS clade | 18 | 60 |
| *E. platanella* group  including RMNH.INS.29651 | 24 | 36 |
| *E. platanella* group  excluding RMNH.INS.29651 | 88 | 100 |
| *E. ornatella* group | 56 | 72 |
| *E. suberis* group | 34 | 96 |
| SUPO clade | 96 | 92 |
| *E. subbimaculella* group | 94 | 88 |
| *E. populella* group | 100 | 100 |
