# Supplementary material for: A Global Phylogeny of Leafmining Ectoedemia Moths (Lepidoptera: Nepticulidae): Exploring Host Plant Family Shifts and Allopatry as Drivers of Speciation
Source: PLoS One. 2015 Mar 18;10(3):e0119586. doi: 10.1371/journal.pone.0119586 (PMC4365004; doi:10.1371/journal.pone.0119586)
Supplement: S4 Table — (DOCX) [file pone.0119586.s004.docx]

**Supporting Information 4 to:**

**A global phylogeny of leafmining *Ectoedemia* moths (Lepidoptera: Nepticulidae): exploring host plant family shifts and allopatry as drivers of speciation.**

Camiel Doorenweerd, Erik J. van Nieukerken & Steph B. J. Menken

PLoS one

**SI4: Supporting information table 4. Identifiers, taxonomy and Genbank accession numbers of the material used in this study.**

| Identifier | Species | 28S | COI-5P | COII | EF1-α | HISTON3 | IDH_NEP |
| --- | --- | --- | --- | --- | --- | --- | --- |
| RMCA.Ent.03177 | *E.* Kenya | - | KM077576 | KM078163 | - | KM078471 | - |
| RMNH.INS.11278 | *E. atricollis* | KM078283 | JN201689.1 | - | JN208708.1 | - | - |
| RMNH.INS.11299 | *E. atricollis* | KM078287 | JN201688.1 | - | JN208707.1 | - | - |
| RMNH.INS.11302 | *E. agrimoniae* | KM078288 | JN201617.1 | - | JN208636.1 | - | - |
| RMNH.INS.11306 | *E. arcuatella* | KM078289 | JN201673.1 | - | JN208691.1 | - | - |
| RMNH.INS.11320 | *E. turbidella* | KM078290 | JN201859.1 | KM078164 | JN208860.1 | - | KM078062 |
| RMNH.INS.11325 | *E. rubivora* | KM078291 | JN201819.1 | - | JN208823.1 | KM078472 | KM078063 |
| RMNH.INS.11329 | *E. subbimaculella* | KM078293 | JN201848.1 | - | JN208850.1 | - | - |
| RMNH.INS.11347 | *E. quinquella* | KM078294 | JN201809.1 | - | JN208814.1 | - | - |
| RMNH.INS.11352 | *E. heringi* | KM078295 | JN201742.1 | - | KM077801 | - | - |
| RMNH.INS.11353 | *E. heringi* | KM078296 | JN201741.1 | KM078165 | JN208757.1 | KM078473 | KM078064 |
| RMNH.INS.11356 | *E. heringi* | KM078297 | JN201740.1 | KM078166 | JN208756.1 | KM078474 | KM078065 |
| RMNH.INS.11407 | *E. suberis* | KM078299 | JN201851.1 | - | JN208852.1 | KM078475 | KM078066 |
| RMNH.INS.11414 | *E. phyllotomella* | KM078300 | JN201789.1 | - | JN208799.1 | - | - |
| RMNH.INS.11416 | *E. phyllotomella* | KM078301 | JN201788.1 | - | JN208798.1 | - | - |
| RMNH.INS.11417 | *E. liechtensteini* | KM078302 | JN201767.1 | - | KM077750 | - | - |
| RMNH.INS.11418 | *E. liechtensteini* | - | JN201766.1 | KM078167 | JN208780.1 | KM078476 | KM078067 |
| RMNH.INS.11534 | *E. erythrogenella* | KM078304 | JN201706.1 | KM078168 | JN208726.1 | KM078477 | - |
| RMNH.INS.11637 | *E. leucothorax* | KM078306 | JN201763.1 | KM078169 | JN208777.1 | KM078478 | - |
| RMNH.INS.11676 | *E. mahalebella* | KM078307 | JN201773.1 | KM078170 | JN208784.1 | KM078479 | - |
| RMNH.INS.11738 | *E. hannoverella* | KM078308 | JN201712.1 | KM078171 | JN208733.1 | - | KM078068 |
| RMNH.INS.11746 | *E. occultella* | KM078309 | JN201782.1 | KM078172 | JN208793.1 | KM078480 | KM078069 |
| RMNH.INS.11761 | *E. argyropeza* | KM078310 | - | - | JN208696.1 | - | - |
| RMNH.INS.11817 | *E. atrifrontella* | KM078315 | KM077633 | - | KM077755 | - | - |
| RMNH.INS.11860 | *E. atrifrontella* | KM078316 | KM077639 | KM078173 | KM077757 | KM078481 | KM078070 |
| RMNH.INS.11865 | *E. heringella* | KM078318 | JN201728.1 | KM078174 | JN208748.1 | KM078482 | KM078071 |
| RMNH.INS.11889 | *E. alnifoliae* | KM078319 | JN201643.1 | KM078175 | JN208663.1 | KM078483 | KM078072 |
| RMNH.INS.11893 | *E. alnifoliae* | KM078320 | JN201642.1 | - | JN208662.1 | - | - |
| RMNH.INS.11896 | *E. haraldi* | KM078321 | JN201716.1 | - | JN208737.1 | - | KM078073 |
| RMNH.INS.11925 | *E. albifasciella* | KM078322 | JN201636.1 | KM078176 | JN208655.1 | KM078484 | KM078074 |
| RMNH.INS.12581 | *E. spinosella* | KM078323 | JN201829.1 | KM078177 | JN208832.1 | - | - |
| RMNH.INS.12585 | *E. spinosella* | KM078324 | JN201828.1 | KM078178 | JN208831.1 | KM078485 | KM078075 |
| RMNH.INS.12587 | *E. rosae* | KM078325 | JN201609.1 | KM078179 | JN208629.1 | KM078486 | KM078076 |
| RMNH.INS.12593 | *E. arcuatella* | KM078327 | JN201671.1 | KM078180 | JN208689.1 | KM078487 | KM078077 |
| RMNH.INS.12613 | *E. agrimoniae* | KM078328 | JN201616.1 | KM078181 | - | KM078488 | KM078078 |
| RMNH.INS.12651 | *E. albifasciella* | KM078330 | JN201634.1 | KM078182 | JN208653.1 | KM078489 | KM078079 |
| RMNH.INS.12705 | *E. suberis* | - | JN201849.1 | - | JN208851.1 | - | - |
| RMNH.INS.12715 | *E. ilicis* | KM078332 | JN201749.1 | KM078183 | JN208763.1 | KM078490 | KM078080 |
| RMNH.INS.12717 | *E. andalusiae* | KM078333 | JN201647.1 | - | JN208666.1 | - | - |
| RMNH.INS.12764 | *E. angulifasciella* | KM078338 | JN201660.1 | KM078184 | JN208679.1 | KM078491 | KM078081 |
| RMNH.INS.12767 | *E. angulifasciella* | KM078341 | JN201657.1 | KM078185 | JN208676.1 | - | KM078082 |
| RMNH.INS.12773 | *E. intimella* | KM078342 | JN201753.1 | - | JN208767.1 | KM078492 | KM078083 |
| RMNH.INS.12778 | *E. minimella* | - | JN201777.1 | - | JN208788.1 | - | - |
| RMNH.INS.12810 | *E. hannoverella* | - | JN201711.1 | - | JN208732.1 | - | - |
| RMNH.INS.12818 | *E. atricollis* | KM078343 | JN201684.1 | KM078186 | JN208703.1 | KM078493 | KM078084 |
| RMNH.INS.12872 | *E. spinosella* | - | JN201827.1 | KM078187 | JN208830.1 | KM078494 | KM078085 |
| RMNH.INS.12900 | *E. pubescivora* | - | JN201804.1 | - | JN208809.1 | - | - |
| RMNH.INS.12987 | *E. minimella* | KM078344 | JN201776.1 | KM078188 | JN208787.1 | KM078495 | KM078086 |
| RMNH.INS.17622 | *E. argyropeza* | - | JN201675.1 | - | JN208693.1 | - | - |
| RMNH.INS.17642 | *E. intimella* | - | JN201752.1 | - | JN208766.1 | - | - |
| RMNH.INS.17677 | *E. rufifrontella* | - | JN201825.1 | KM078189 | JN208828.1 | KM078497 | KM078088 |
| RMNH.INS.17747 | *E. terebinthivora* | KM078345 | JN201853.1 | KM078190 | JN208854.1 | KM078498 | KM078089 |
| RMNH.INS.17759 | *E. terebinthivora* | KM078346 | JN201852.1 | KM078191 | JN208853.1 | KM078499 | KM078090 |
| RMNH.INS.17774 | *E. argyropeza* | KM078347 | JN201674.1 | KM078192 | JN208692.1 | - | KM078091 |
| RMNH.INS.17805 | *E. agrimoniae* | - | JN201615.1 | KM078193 | JN208635.1 | - | KM078092 |
| RMNH.INS.17827 | *E. klimeschi* | - | JN201760.1 | KM078194 | JN208774.1 | KM078500 | KM078093 |
| RMNH.INS.17828 | *E. klimeschi* | KM078348 | JN201759.1 | - | JN208773.1 | KM078501 | KM078094 |
| RMNH.INS.17833 | *E. turbidella* | - | JN201857.1 | KM078195 | JN208858.1 | KM078502 | KM078095 |
| RMNH.INS.17851 | *E. rubivora* | - | JN201811.1 | - | JN208815.1 | KM078503 | KM078096 |
| RMNH.INS.17868 | *E. quinquella* | KM078349 | JN201807.1 | KM078196 | JN208812.1 | KM078504 | - |
| RMNH.INS.17896 | *E. spiraeae* | KM078350 | JN201831.1 | - | JN208834.1 | KM078506 | - |
| RMNH.INS.17902 | *E. cerviparadisicola* | KM078351 | JN201607.1 | - | JN208628.1 | KM078507 | KM078097 |
| RMNH.INS.17989 | *E. Annamocarya_Vietnam* | KM078352 | JN201598.1 | KM078197 | JN208621.1 | - | KM078098 |
| RMNH.INS.18228 | *E. quadrinotata* | KM078354 | KM077635 | - | KM077756 | - | - |
| RMNH.INS.18237 | *E. occultella* | KM078355 | KM077691 | KM078198 | KM077787 | KM078508 | KM078099 |
| RMNH.INS.18254 | *E. Quercus_velutina_USA* | KM078356 | KM077679 | - | KM077778 | - | - |
| RMNH.INS.18260 | *E. nyssaefoliella* | KM078357 | KM077703 | KM078199 | KM077794 | KM078509 | - |
| RMNH.INS.18265 | *E. virgulae* | KM078358 | KM077702 | - | KM077793 | - | - |
| RMNH.INS.18282 | *E. clemensella* | KM078359 | KM077729 | KM078200 | KM077804 | KM078510 | KM078100 |
| RMNH.INS.18285 | *E. clemensella* | KM078360 | KM077605 | - | KM077744 | - | - |
| RMNH.INS.18286 | *E. ulmella* | KM078361 | - | KM078201 | - | KM078511 | KM078101 |
| RMNH.INS.18288 | *E. ulmella* | KM078362 | KM077656 | KM078202 | KM077765 | KM078512 | KM078102 |
| RMNH.INS.18297 | *E. trinotata* | KM078363 | KM077719 | KM078203 | KM077800 | KM078513 | KM078103 |
| RMNH.INS.18333 | *E. platanella* | KM078365 | KM077598 | KM078204 | KM077741 | - | KM078104 |
| RMNH.INS.18358 | *E. platanella* | KM078366 | KM077592 | - | KM077739 | - | - |
| RMNH.INS.18373 | *E. similella* | KM078368 | KM077657 | KM078205 | KM077766 | KM078514 | KM078105 |
| RMNH.INS.18389 | *E. rubifoliella* | KM078369 | KM077619 | KM078206 | KM077748 | KM078515 | - |
| RMNH.INS.18410 | *E. rubifoliella* | KM078371 | KM077696 | KM078207 | KM077789 | - | KM078106 |
| RMNH.INS.18501 | *E. spiraeae* | KM078373 | KM077727 | KM078208 | KM077803 | KM078516 | - |
| RMNH.INS.18505 | *E. populella* | KM078374 | KM077710 | KM078209 | KM077797 | - | KM078107 |
| RMNH.INS.18506 | *E. virgulae* | KM078375 | KM077733 | - | KM077805 | - | - |
| RMNH.INS.18517 | *E. virgulae* | KM078376 | KM077622 | - | KM077749 | - | - |
| RMNH.INS.18556 | *E. virgulae* | KM078377 | KM077626 | KM078210 | KM077751 | - | KM078108 |
| RMNH.INS.18557 | *E. quadrinotata* | KM078378 | KM077697 | KM078211 | KM077790 | - | KM078109 |
| RMNH.INS.18644 | *E. virgulae* | KM078380 | KM077671 | KM078212 | KM077772 | KM078517 | KM078110 |
| RMNH.INS.18664 | *E. Quercus_rubra_USA* | - | KM077661 | KM078213 | - | - | KM078111 |
| RMNH.INS.18857 | *E. heringi* | KM078381 | KM077684 | KM078214 | KM077784 | KM078518 | KM078112 |
| RMNH.INS.23424 | *E. haraldi* | KM078382 | JN201715.1 | - | JN208736.1 | - | - |
| RMNH.INS.23555 | *E. longicaudella* | KM078383 | KM077582 | - | KM077736 | - | - |
| RMNH.INS.23602 | *E. erythrogenella* | KM078385 | JN201703.1 | KM078215 | JN208723.1 | KM078519 | KM078113 |
| RMNH.INS.23629 | *E. rufifrontella* | KM078386 | JN201823.1 | KM078216 | JN208827.1 | KM078520 | - |
| RMNH.INS.23661 | *E. subbimaculella* | KM078387 | JN201838.1 | KM078217 | JN208841.1 | KM078521 | KM078114 |
| RMNH.INS.23662 | *E. haraldi* | KM078388 | JN201714.1 | KM078218 | JN208735.1 | KM078522 | - |
| RMNH.INS.23667 | *E. ilicis* | KM078389 | JN201748.1 | KM078219 | JN208762.1 | KM078523 | KM078115 |
| RMNH.INS.23674 | *E. heringella* | KM078390 | JN201722.1 | - | JN208743.1 | - | - |
| RMNH.INS.23677 | *E. reichli* | KM078392 | JN201810.1 | KM078220 | - | KM078524 | - |
| RMNH.INS.23684 | *E. liebwerdella* | KM078394 | JN201764.1 | KM078221 | JN208778.1 | - | KM078116 |
| RMNH.INS.23685 | *E. longicaudella* | KM078395 | JN201768.1 | KM078222 | KM077769 | KM078525 | KM078117 |
| RMNH.INS.23693 | *E. pseudoilicis* | KM078396 | JN201800.1 | - | - | - | - |
| RMNH.INS.23720 | *E. hendrikseni* | KM078397 | JN201721.1 | KM078223 | JN208742.1 | KM078526 | - |
| RMNH.INS.23723 | *E. nrcanutus* | KM078398 | JN201690.1 | KM078224 | JN208709.1 | KM078527 | KM078118 |
| RMNH.INS.23727 | *E. canutus* | KM078399 | JN201604.1 | KM078225 | JN208625.1 | KM078528 | KM078119 |
| RMNH.INS.23730 | *E. heckfordi* | KM078400 | JN201719.1 | KM078226 | JN208740.1 | KM078529 | KM078120 |
| RMNH.INS.23741 | *E. rosae* | KM078401 | JN201603.1 | KM078227 | JN208624.1 | KM078530 | KM078121 |
| RMNH.INS.23752 | *E. pubescivora* | KM078402 | JN201801.1 | KM078228 | JN208808.1 | KM078531 | KM078122 |
| RMNH.INS.23762 | *E. albifasciella* | KM078403 | JN201602.1 | - | JN208623.1 | - | - |
| RMNH.INS.23820 | *E. amani* | KM078404 | JN201644.1 | KM078229 | JN208664.1 | - | KM078123 |
| RMNH.INS.23825 | *E. mahalebella* | KM078405 | JN201769.1 | KM078230 | JN208781.1 | KM078532 | KM078124 |
| RMNH.INS.23828 | *E. hendrikseni* | KM078406 | JN201720.1 | - | JN208741.1 | - | - |
| RMNH.INS.23829 | *E. phaeolepis* | KM078407 | JN201787.1 | KM078231 | JN208797.1 | - | KM078125 |
| RMNH.INS.23831 | *E. pseudoilicis* | KM078408 | JN201799.1 | KM078232 | JN208807.1 | KM078533 | - |
| RMNH.INS.23834 | *E. leucothorax* | KM078409 | JN201762.1 | - | JN208776.1 | - | - |
| RMNH.INS.23835 | *E. contorta* | KM078410 | JN201699.1 | - | JN208718.1 | KM078534 | - |
| RMNH.INS.23836 | *E. cerris* | KM078411 | JN201695.1 | KM078233 | JN208714.1 | KM078535 | - |
| RMNH.INS.23839 | *E. caradjai* | KM078412 | JN201692.1 | KM078234 | JN208711.1 | - | - |
| RMNH.INS.23840 | *E. gilvipennella* | KM078413 | JN201708.1 | KM078235 | JN208728.1 | KM078536 | KM078126 |
| RMNH.INS.23842 | *E. algeriensis* | KM078414 | JN201640.1 | - | JN208660.1 | - | - |
| RMNH.INS.23843 | *E. subbimaculella* | KM078415 | JN201835.1 | KM078236 | JN208838.1 | KM078537 | KM078127 |
| RMNH.INS.23844 | *E. phaeolepis* | - | JN201786.1 | - | JN208796.1 | - | - |
| RMNH.INS.23845 | *E. coscoja* | KM078416 | JN201701.1 | - | JN208720.1 | KM078538 | - |
| RMNH.INS.23848 | *E. heckfordi* | KM078417 | JN201718.1 | KM078237 | JN208739.1 | KM078539 | KM078128 |
| RMNH.INS.23870 | *E. preisseckeri* | KM078419 | JN201797.1 | KM078238 | JN208804.1 | KM078540 | - |
| RMNH.INS.23872 | *E. caradjai* | KM078420 | JN201691.1 | - | JN208710.1 | - | - |
| RMNH.INS.23873 | *E. andalusiae* | KM078421 | JN201646.1 | KM078239 | JN208665.1 | KM078541 | - |
| RMNH.INS.23875 | *E. aegilopidella* | - | JN201614.1 | - | JN208634.1 | KM078542 | - |
| RMNH.INS.23876 | *E. algeriensis* | KM078422 | JN201639.1 | KM078240 | JN208659.1 | KM078543 | KM078129 |
| RMNH.INS.23878 | *E. gilvipennella* | KM078423 | JN201707.1 | - | JN208727.1 | - | - |
| RMNH.INS.23880 | *E. cerris* | - | JN201694.1 | - | JN208713.1 | - | - |
| RMNH.INS.23881 | *E. contorta* | - | JN201698.1 | - | JN208717.1 | - | - |
| RMNH.INS.23883 | *E. alnifoliae* | - | JN201641.1 | KM078241 | JN208661.1 | KM078544 | - |
| RMNH.INS.23884 | *E. erythrogenella* | - | JN201702.1 | - | JN208722.1 | KM078545 | - |
| RMNH.INS.23886 | *E. populella* | - | JN201794.1 | - | JN208802.1 | KM078546 | - |
| RMNH.INS.23887 | *E. arisi* | - | JN201680.1 | KM078242 | JN208699.1 | KM078547 | - |
| RMNH.INS.23888 | *E. picturata* | - | JN201790.1 | - | JN208800.1 | KM078548 | - |
| RMNH.INS.23891 | *E. picturata* | - | JN201792.1 | - | JN208801.1 | KM078549 | - |
| RMNH.INS.23892 | *E.Rubus_Vietnam_Fansipan* | KM078424 | JN201613.1 | KM078243 | JN208632.1 | KM078550 | - |
| RMNH.INS.23893 | *E. TamDao* | KM078425 | JN201612.1 | KM078244 | JN208631.1 | KM078551 | - |
| RMNH.INS.23894 | *E. Rubus_Borneo* | KM078426 | JN201611.1 | - | JN208630.1 | KM078552 | KM078130 |
| RMNH.INS.23896 | *E. ornatella* | KM078427 | JN201784.1 | - | - | - | KM078131 |
| RMNH.INS.23897 | *E. christopheri* | KM078428 | - | - | JN208716.1 | KM078553 | - |
| RMNH.INS.23899 | *E. ivinskisi* | KM078429 | JN201758.1 | KM078245 | JN208771.1 | KM078554 | - |
| RMNH.INS.23902 | *E. Carpinus_Vietnam* | KM078430 | JN201610.1 | KM078246 | KM077781 | - | - |
| RMNH.INS.23912 | *E. chasanella* | KM078431 | JN201697.1 | - | JN208715.1 | KM078555 | - |
| RMNH.INS.23987 | *E. expeditionis* | KM078432 | JN201855.1 | KM078247 | JN208856.1 | - | KM078132 |
| RMNH.INS.23988 | *E. tersiusi* | KM078433 | JN201854.1 | - | JN208855.1 | - | - |
| RMNH.INS.23989 | *E. Namibia* | - | JN201600.1 | - | - | KM078556 | - |
| RMNH.INS.24061 | *E. canutus* | KM078435 | KM077599 | - | KM077742 | - | - |
| RMNH.INS.24063 | *E. ornatella* | KM078436 | KM077652 | - | - | KM078557 | - |
| RMNH.INS.24064 | *E. TaiwanEvN4064* | KM078437 | KM077580 | - | KM077735 | KM078558 | - |
| RMNH.INS.24119 | *E. piperella* | - | KM077714 | KM078249 | - | - | - |
| RMNH.INS.24179 | *E.Rubus_Vietnam_Fansipan* | - | KM077596 | - | - | KM078561 | - |
| RMNH.INS.24213 | *E. Nyssa_sp_n_USA* | KM078441 | KM077614 | KM078252 | KM077746 | KM078562 | KM078133 |
| RMNH.INS.24214 | *E. nyssaefoliella* | KM078442 | KM077602 | KM078253 | KM077743 | - | KM078134 |
| RMNH.INS.24295 | *E. argyropeza* | - | KM077720 | - | - | - | - |
| RMNH.INS.24335 | *E. similella* | - | KM077660 | - | - | - | - |
| RMNH.INS.24337 | *E. Nyssa_sp_n_USA* | - | KM077731 | - | - | - | - |
| RMNH.INS.24351 | *E. Annamocarya_Vietnam* | - | KM077571 | - | - | - | - |
| RMNH.INS.24395 | *E. virgulae* | - | KM077638 | KM078254 | - | KM078563 | - |
| RMNH.INS.24397 | *E. trinotata* | - | KM077611 | KM078255 | - | KM078564 | KM078135 |
| RMNH.INS.29205 | *E. Prunus_Korea* | KM078443 | KM077646 | KM078256 | KM077760 | KM078565 | KM078136 |
| RMNH.INS.29211 | *E. olvina* | KM078444 | KM077650 | KM078257 | KM077763 | KM078566 | KM078137 |
| RMNH.INS.29215 | *E. Sorbus_Korea* | KM078445 | KM077698 | KM078258 | KM077791 | KM078567 | KM078138 |
| RMNH.INS.29221 | *E. Prunus_Korea* | KM078446 | KM077648 | KM078259 | KM077761 | KM078568 | KM078139 |
| RMNH.INS.29226 | *E. Ulmus_Korea* | KM078447 | KM077642 | KM078260 | KM077758 | KM078569 | KM078140 |
| RMNH.INS.29235 | *E. olvina* | KM078448 | KM077673 | KM078261 | KM077774 | KM078570 | KM078141 |
| RMNH.INS.29256 | *E. pilosae* | KM078449 | KM077709 | KM078262 | KM077796 | KM078571 | KM078142 |
| RMNH.INS.29257 | *E. pilosae* | KM078450 | KM077662 | KM078263 | - | KM078572 | - |
| RMNH.INS.29272 | *E. Rubus_alceifolius_Tw* | KM078451 | KM077717 | KM078264 | KM077798 | KM078573 | KM078143 |
| RMNH.INS.29329 | *E.Rubus_croceacanthus_Tw* | KM078452 | KM077649 | KM078265 | KM077762 | KM078574 | KM078144 |
| RMNH.INS.29341 | *E. Rubus_trianthus_Tw* | KM078453 | KM077680 | KM078266 | KM077779 | KM078575 | KM078145 |
| RMNH.INS.29353 | *E. Pourthiaea_Taiwan* | KM078454 | KM077630 | KM078267 | KM077754 | KM078576 | KM078146 |
| RMNH.INS.29358 | *E. arisi* | KM078455 | KM077681 | KM078268 | KM077780 | KM078577 | KM078147 |
| RMNH.INS.29364 | *E. Acer_Taiwan* | KM078456 | KM077659 | KM078269 | KM077767 | KM078578 | KM078148 |
| RMNH.INS.29382 | *E. Carpinus_Taiwan* | KM078457 | KM077609 | KM078270 | KM077745 | KM078579 | KM078149 |
| RMNH.INS.29383 | *E. Carpinus_Taiwan* | KM078458 | KM077677 | KM078271 | KM077776 | KM078580 | KM078150 |
| RMNH.INS.29385 | *E. Quercus_variabilis_Tw* | KM078459 | KM077645 | KM078272 | KM077759 | KM078581 | KM078151 |
| RMNH.INS.29386 | *E. Quercus_spinosa_Tw* | KM078460 | KM077718 | KM078273 | KM077799 | KM078582 | KM078152 |
| RMNH.INS.29391 | *E. Quercus_tatakaensis_Tw* | KM078461 | KM077628 | KM078274 | KM077753 | KM078583 | KM078153 |
| RMNH.INS.29402 | *E. Pourthiaea_Taiwan* | KM078462 | KM077616 | KM078275 | KM077747 | KM078584 | KM078154 |
| RMNH.INS.29404 | *E. Rosa_Taiwan* | KM078463 | KM077678 | KM078276 | KM077777 | - | KM078155 |
| RMNH.INS.29415 | *E.* *Rosa_Taiwan* | KM078464 | KM077664 | KM078277 | KM077768 | KM078585 | KM078156 |
| RMNH.INS.29454 | *E. Rubus_Taiwan* | KM078465 | KM077627 | - | KM077752 | KM078586 | KM078157 |
| RMNH.INS.29480 | *E.Rubus_croceacanthus_Tw* | KM078466 | KM077651 | - | KM077764 | KM078587 | KM078158 |
| RMNH.INS.29531 | *E. Quercus_tatakaensis_Tw* | KM078467 | KM077699 | KM078278 | KM077792 | KM078588 | KM078159 |
| RMNH.INS.29536 | *E.* *Quercus_variabilis_Tw* | KM078468 | KM077573 | KM078279 | - | KM078589 | KM078160 |
| RMNH.INS.29547 | *E. Lithocarpus_Taiwan* | KM078469 | KM077666 | KM078280 | KM077770 | KM078590 | KM078161 |
| RMNH.INS.29651 | *E. Platanus_wrightii_AZ* | KM078470 | KM077669 | KM078281 | KM077771 | KM078591 | KM078162 |
